# Genetic Deletion of *miR-430* Disrupts Maternal-Zygotic Transition and Embryonic Body Plan

**DOI:** 10.3389/fgene.2020.00853

**Published:** 2020-08-04

**Authors:** Yun Liu, Zeyao Zhu, Idy H. T. Ho, Yujian Shi, Jianzhen Li, Xia Wang, Matthew T. V. Chan, Christopher H. K. Cheng

**Affiliations:** ^1^School of Biomedical Sciences, The Chinese University of Hong Kong, Hong Kong, China; ^2^Shenzhen Zelong Biological Technology Limited Cooperation, Shenzhen, China; ^3^Department of Anatomy, Histology and Developmental Biology, School of Basic Medical Sciences, Shenzhen University, Shenzhen, China; ^4^Department of Anesthesia and Intensive Care, Prince of Wales Hospital, Faculty of Medicine, The Chinese University of Hong Kong, Hong Kong, China; ^5^College of Life Sciences, Northwest Normal University, Lanzhou, China

**Keywords:** small regulatory RNAs, miR-430, genome editing, gastrulation, cell fate

## Abstract

*MiR-430* is considered an important regulator during embryonic development, but genetic loss-of-function study is still lacking. Here we demonstrated that genetic deletion of the *miR-430* cluster resulted in developmental defects in cell movement, germ layer specification, axis patterning and organ progenitor formation in zebrafish. Transcriptome analysis indicated that the maternally provided transcripts were not properly degraded whereas the zygotic genome expressed genes were not fully activated in the *miR-430* mutants. We further found that a reciprocal regulatory loop exists between *miR-430* and maternally provided transcripts: the maternally provided transcripts (*Nanog*, *Dicer1*, *Dgcr8*, and *AGOs*) are required for *miR-430* biogenesis and function, whereas *miR-430* is required for the clearance of these maternally provided transcripts. These data provide the first genetic evidence that *miR-430* is required for maternal-zygotic transition and subsequent establishment of embryonic body plan.

## Introduction

MicroRNAs (miRNAs) are ∼22-nucleotide (nt) non-coding RNAs that repress mRNA expression post-transcriptionally (Bartel, 2009). Transcribed by RNA polymerase II, the primary miRNA transcripts are processed by *Dgcr8* and RNase III enzyme Drosha into 70-nt precursors which are further processed into 21–23-nt mature microRNAs by *Dicer* (Kim et al., 2009). One strand of the mature microRNAs is loaded into argonaute (AGO) protein to recognize target mRNAs by pairing with miRNA binding sites in the 3′ untranslated region (UTR) (Kim et al., 2009). AGO proteins further recruit other factors that could induce translational repression, mRNA deadenylation and mRNA decay (Fabian et al., 2010). One miRNA can modulate hundreds of mRNA targets and more than 60% of human protein-coding genes are considered targets for miRNAs (Friedman et al., 2009).

In mice, zygotic deletion of *Dgcr8* or *Dicer* leads to embryonic arrest shortly after implantation (Bernstein et al., 2003; Wang et al., 2007). In zebrafish, *Dicer1* transcripts are maternally provided and maternal-zygotic dicer (MZ*dicer*) mutant exhibits cell movement defects at the onset of gastrulation (Wienholds et al., 2003; Giraldez et al., 2005, 2006). Recently, we have successfully generated zebrafish maternal-zygotic *dgcr8* (MZ*dgcr8*) mutant and found that MZ*dgcr8* and MZ*dicer* share similar phenotypes (Liu et al., 2017; Zhu et al., 2020), suggesting that the canonical miRNAs play important roles in early development. Interestingly, *miR-430* could rescue some developmental defects of MZ*dicer* and MZ*dgcr8* mutants, suggesting that suggesting that *miR-430* might be the key effector of the miRNA pathway in early embryogenesis (Giraldez et al., 2005, 2006; Liu et al., 2017).

The maternally provided transcripts play central roles in the regulation of early embryonic development (Lee et al., 2013; Leichsenring et al., 2013). In zebrafish, the maternally provided transcripts *Pou5f1*, *Nanog*, and *Sox19b* are required for zygotic genome activation (ZGA) (Lee et al., 2013; Leichsenring et al., 2013). *MiR-430* is among the first-wave of zygotic genome expressed genes activated by *Nanog* (Lee et al., 2013). Using MZ*dicer* mutants, *miR-430* was suggested to destabilize hundreds of maternal transcripts after ZGA (Giraldez et al., 2006). Moreover, *miR-430* was also found to regulate the nodal pathway and other developmental pathways during early development (Mishima et al., 2006; Choi et al., 2007; Rosa et al., 2009; Staton et al., 2011; Wylie et al., 2014; van Boxtel et al., 2015; Takacs and Giraldez, 2016). However, genetic loss-of-function study of *miR-430* is still lacking. In this study, we generated *miR-430*-deficient zebrafish and found that *miR-430* is required for maternal-zygotic transition (MZT) and establishment of embryonic body plan.

## Materials and Methods

### Zebrafish Husbandry

AB zebrafish were maintained at 28°C in the zebrafish facility of Sun Yat-sen University and the Chinese University of Hong Kong. All animal experiments were conducted in accordance with the guidelines and approval of the respective Animal Research and Ethics Committees.

### Generation of *miR-430*^–/–^ Mutant Line

The method for deleting the *miR-430* gene cluster was reported in previous studies (Lei et al., 2012; Liu et al., 2013, 2014). Germline transmission of *miR-430* deletion was reported in our previous study (Liu et al., 2013). To obtain homozygous mutants, heterozygous mutants of the same mutation were obtained and self-crossed.

### Morphological Analysis and Rescue Experiments

To analyze the phenotypic consequences of *miR-430* loss-of-function, embryos from incrosses of *miR-430* heterozygote adults were collected and genotyped by PCR (Supplementary Figure S1). Because the *miR-430*^–/–^ mutants showed developmental delay, embryos of the same developmental stages or at same time points were photographed.

To perform rescue experiments, *miR-430* mimics were synthesized as described (Giraldez et al., 2005, 2006). For phenotypic rescue, *miR-430* mimics (10 pg/embryo) or mismatched *miR-430b* mimics were injected into one-cell stage embryos and the phenotypic changes were recorded on a stereomicroscope (Olympus). For miRNA rescue efficiency analysis, marker gene analysis and transcriptome sequencing, *miR-430*^–/–^ embryos produced by incrosses of the rescued *miR-430*^–/–^ adult fish were used.

### Whole-Mount *in situ* Hybridization

The antisense probes of marker genes were prepared using the DIG RNA Labeling Kit (Roche, United States). The probe information was provided in the Supplementary Table S1. Whole mount *in situ* hybridization was performed as described (Li et al., 2014). The embryos of similar developmental stages were collected and the ratios of the affected embryos were calculated.

### Identification of *miR-430* Target Genes

Total RNA was isolated from shield stage embryos of WT, *miR-430*^–/–^ and rescued groups with the RNeasy Mini Kit (QIAGEN). Deep-sequencing of total RNA (ribosomal RNA minus) was performed on an Illumina HiSeq2000 platform at BGI (Shenzhen, China). Approximately 5 Gb of raw reads were generated for each sample. The fragments per kilobase million (FPKM) values of the mapped genes were obtained using the Cufflink software (Trapnell et al., 2012). The differentially expressed genes were identified by the defined thresholds of fold changes and *p* value. The *miR-430* binding sites were predicted by TargetScanFish 6.0^1^ (Ulitsky et al., 2012). Gene ontology (GO) analysis was performed by the DAVID software^2^ (Huang et al., 2009). The reported transcriptome data in a previous study (Yang et al., 2013) were used to examine gene expression profiles during early embryonic development in zebrafish.

### Q-PCR Analysis of mRNA Expression

Total RNAs were isolated from zebrafish embryos of shield stage using the RNeasy Mini Kit (QIAGEN). Complementary DNA was synthesized using the PrimeScript RT Reagent Kit (TAKARA). Real-time Q-PCR was performed on an ABI PRISM 7900 Sequence Detection System (Applied Biosystems) using the SYBR Green I Kit (Applied Biosystem). The mRNA transcript levels were normalized against the ef1α transcript level. Primers used in this study are listed in Supplementary Table S1.

### *Lefty2* (*lft2*) Knockdown Study

The MO sequence for *lft2* knockdown was designed as reported (Feldman et al., 2002). The *lft2* MO or control MO (8 ng/embryo) were injected into the *miR-430*^–/–^ mutants. The embryos were collected at 75% epiboly and 36 h post fertilization (36 hpf) for marker gene expression analysis.

### *MiR-430* Mediated Clearance of Maternally Provided Dicer1

The DNA sequence of *Dicer*-3′UTR-WT or *Dicer*-3′UTR-mut were amplified by PCR and cloned into pCS2-3XIPT-*miR-430* plasmid by *Xho*I and *Xba*I (Giraldez et al., 2005). GFP sensor mRNAs were transcribed using the mMESSAGE mMACHINE SP6 kit (Ambion) and purified using the RNeasy Mini Kit (QIAGEN). One hundred nanogram of mRNA was injected into a *miR-430*^–/–^ mutant or WT embryo at one cell stage. The GFP signal was analyzed on a fluorescent microscope (Olympus) at 6 hpf.

### Statistical Analyses

The Q-PCR data are expressed as the mean values ± SEM. Statistical analyses were performed using *t*-test or one-way ANOVA followed by Tukey’s Multiple Comparison Test (GraphPad). The enrichment analyses were performed using Chi-squared test (Microsoft Excel software). Results were considered statistically significant at *P* < 0.05.

## Results

### Targeted Genetic Deletion of *miR-430*

**FIGURE 1:**
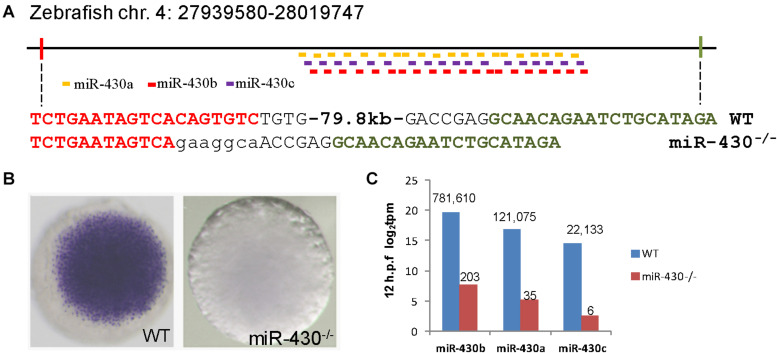
Targeted deletion of *miR-430* in zebrafish. **(A)** Genetic deletion of zebrafish *miR-430* cluster using TALENs. A 79.8 kb genomic fragment containing the zebrafish *miR-430* was deleted using two pairs of TALENs. The TALEN binding sites were shown in colors and the inserted nucleotides were shown in lower case letters. *MiR-430* isoforms were indicated by colored short lines. **(B)** *In situ* hybridization detection of *pri-mir-430* expression in the WT and *miR-430*^–/–^ mutant embryos at 4 hpf. **(C)** Expression profiles of mature *miR-430* isoforms in the WT and *miR-430*^–/–^ mutant embryos (12 hpf) by small RNA deep sequencing. The numbers of normalized reads were indicated. TPM, transcripts per kilobase million.

To investigate the functional roles of *miR-430* during early embryonic development, we assembled two pairs of TALENs to delete a ∼80 kb genomic fragment containing the zebrafish *miR-430* cluster (Figure 1A) (Liu et al., 2013). This deleted region mainly contains repeated sequences with no recognizable functional genes other than miR-430. The miR-430 cluster is the largest identified miRNA cluster which contains 57 miRNAs with the same miRNA seed sequence in zebrafish (Figure 1A). In this study, we have characterized the phenotypes of *miR-430* homozygous mutants. Whole mount *in situ* hybridization analysis indicated that the primary *miR-430* transcript was abundantly expressed in the WT (4 hpf) but not in the *miR-430* mutants (Figure 1B). Moreover, the mature *miR-430*s were the most abundantly expressed miRNAs in the 6-somite WT embryos (92% of total miRNA reads) and decreased about 4,000 folds in the *miR-430*^–/–^ mutants (Figure 1C). These data indicated that we have successfully established the loss-of-function model of the *miR-430* cluster in zebrafish.

### *MiR-430* Is Required for the Establishment of Embryonic Body Plan

**FIGURE 2:**
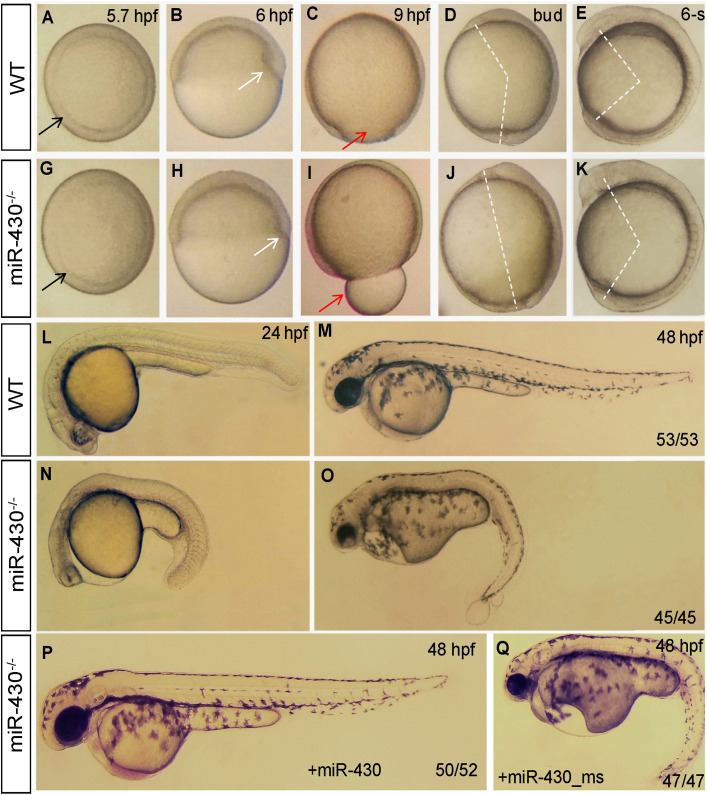
Morphological defects in the *miR-430*-deficient embryos. **(A–O)** The morphology of WT and *miR-430*-deficient embryos. The phenotypes are fully penetrant. Formation of the germ ring (black arrows) and shield (white arrows) were delayed in the *miR-430*^–/–^ mutants **(A,B,F,G)**. The yolk was not enclosed by epiboly in the mutant at 9 hpf **(C,H)**. The extension of the anterior-posterior body axis was reduced in the *miR-430* mutants **(D,E,I,J)**. The *miR-430* mutants had no brain vesicles and lacked heart tissue and circulation **(K,M)**. Tail blisters were observed in the mutant at 48 hpf **(L,N)**. The *miR-430* mimics but not the mismatched *miR-430* with 2-nucleotide alterations could rescue the defects observed in the *miR-430* mutants **(O,P)**. The ratios of observed phenotypes were indicated.

We then analyzed the phenotypic consequences of *miR-430* deletion. Compared to WT embryos, the *miR-430*^–/–^ mutants exhibited developmental delay since the germ ring stage (5.7 hpf; Figures 2A,F). The formation of shield at the dorsal site was reduced at 6 hpf (Figures 2B,G). The mutant embryos exhibited a longer animal-vegetal axis and the yolk was not completely covered by cell sheet at the end of epiboly cell movements (Figures 2C,H). The extension of the anterior-posterior body axis was reduced in the mutant (Figures 2D,E,I,J). The regionalization of the brain was disrupted at 24 hpf (Figures 2K,M). No heart beat or circulation can be observed but tail blister was developed in the mutant at 48 hpf (Figures 2L,N). All mutant died at around 5 days post fertilization (dpf) with a swollen body cavity (*n* = 500) (Supplementary Figure S2).

To test whether the observed phenotypes were due to the loss of function of *miR-430*, we then performed rescue experiments using *miR-430* duplexes (8, 9). Injection of *miR-430* duplexes but not the mismatched mimics efficiently rescued the mutant phenotypes (Figures 2O,P). Some of rescued embryos even survived to adulthood. We further crossed the rescued *miR-430*^–/–^ mutant adult fish to produce the MZ*miR-430* mutants. The MZ*miR-430* embryos had the same phenotypes as the Z*miR-430* mutants. Further rescue of the MZ*miR-430* embryos indicated that eight of the 52 rescued embryos survived to adulthood. These data indicated that *miR-430* is mainly functional during early embryonic development.

**FIGURE 3:**
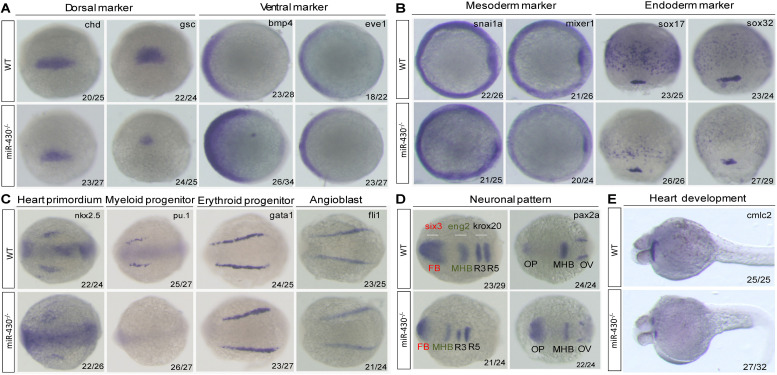
Marker gene analysis. Marker gene expression of the WT and *miR-430*^–/–^ mutant was analyzed at shield stage **(A)**, 75% epiboly **(B)**, 6-somite **(C,D)**, and 36 hpf **(E)**. Marker genes of axis formation **(A)**, germ layer formation **(B)**, organ progenitor formation, **(C)** neuronal pattern **(D)**, and heart formation **(E)** were analyzed in the *miR-430*^–/–^ mutants. The ratios of the observed phenotypes were indicated. FB, forebrain; MHB, midbrain hindbrain boundary; OV, otic vesicle; OP, optic placode; R3, rhombomere 3; R5, rhombomere 5.

We next analyzed the *miR-430*^–/–^ phenotypes using marker genes. In the mutant embryos, expression of the dorsal mesoderm markers (*chd* and *gsc*) were reduced and expression domain of the ventral markers (*eve1* and *bmp4*) were expanded (Figure 3A). The mesoderm (*snai1a* and *mixer1*) and endoderm markers (*sox17* and *sox32*) were down-regulated (Figure 3B). The erythroid progenitor marker (*gata1*) was expanded but the myeloid progenitor marker (*pu.1*) was absent (Figure 3C). The cardiac progenitors (*nkx2.5*) were reduced and the cardiocytes (*cmlc2*) failed to migrate to the middle line, resulted in cardiac bifida (Figures 3C,E). The angioblast marker *fli-1* was specified but the vascular system was disrupted at later stages (Figure 3C and Supplementary Figure S3). In the neuronal system, the expression of the mid-hind boundary domain markers (*eng2* and *pax2a*) was decreased. The forebrain region was reduced (*six3*) and the optic placode was expanded (*pax2a*) (Figure 3D). Consistent with the morphological observations, the anterior neural plate was reduced anterior-posteriorly but expanded medio-laterally (Figure 3D). These results indicated that *miR-430* is required for axis patterning, germ layer specification, organ progenitor formation and cell movements, thus contributing to the establishment of embryonic body plan (Schier and Talbot, 2005).

### Identification of *miR-430* Targets

**FIGURE 4:**
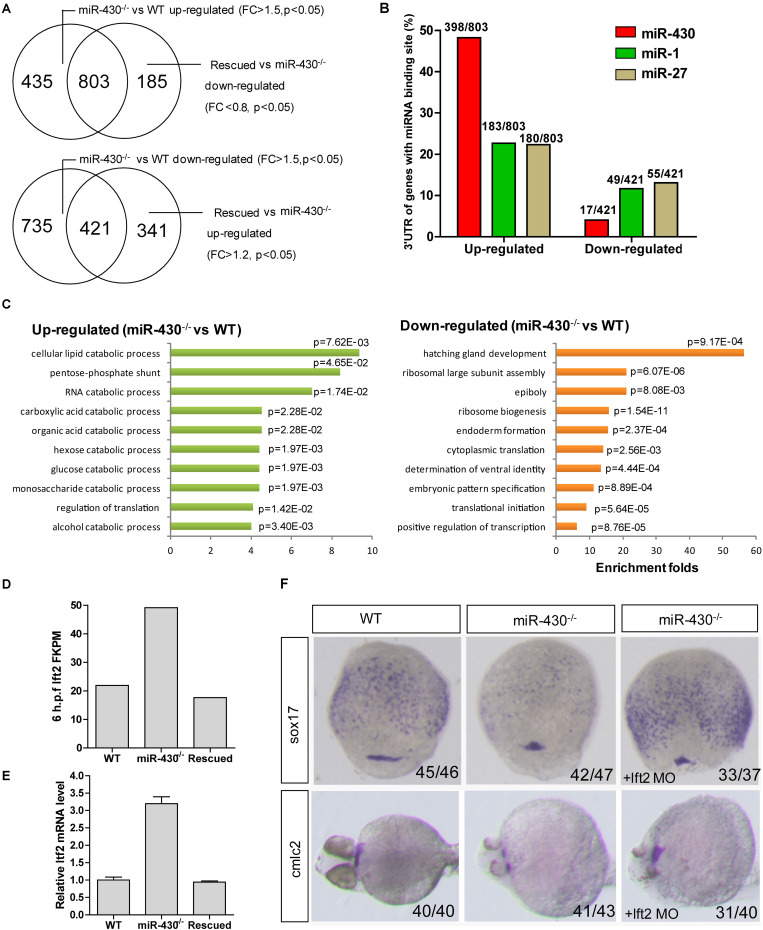
Transcriptome analysis. Total mRNAs of embryos from WT, *miR-430*^–/–^ mutant and rescued groups were collected at shield stage for transcriptome sequencing. **(A)** Identification of *miR-430*-regulated genes. **(B)** *MiR-430* binding site analysis. The ratios indicate the numbers of genes with canonical sites of the indicated miRNA in their 3′UTR/total number of analyzed genes. The miRNA binding sites were predicted by TargetScanFish 6.2. **(C)** GO analysis of the *miR-430* regulated genes, performed by the DAVID system. **(D)** The expression of *lft2* in the transcriptome of the WT, *miR-430*^–/–^ and rescued embryos. **(E)** Q-PCR analysis of *lft2* mRNA expression levels in the WT, *miR-430*^–/–^ and rescued group. Data are expressed as mean values ± S.E.M (*n* = 4). **(F)** MO knockdown of *lft2* partially rescued the *miR-430*-deficient phenotypes. The embryos were collected at 75% epiboly and 36 hpf for WISH analysis of marker gene expression.

To identify *miR-430* targets, we collected the WT, *miR-430*^–/–^ and rescued embryos at shield stage and performed transcriptome sequencing (Supplementary Table S2). The transcriptome data were validated by Q-PCR (Supplementary Figure S4). We reasoned that the *miR-430* regulated genes would be up-regulated in the *miR-430*^–/–^ mutants but down-regulated in the rescued embryos (*miR-430*^–/–^ + miR-430 mimics) and vice versa. We have identified 803 up-regulated genes and 421 down-regulated genes (*miR-430*^–/–^ VS WT, fold change > 1.5, *p* < 0.05; rescued VS *miR-430*^–/–^, fold change < 0.8, *p* < 0.05) (Figure 4A and Supplementary Table S2). About half of the up-regulated genes possess canonical *miR-430* binding sites (8er, 7er-m8, and 7er-1A) in their 3′UTR (1) (Figure 4B and Supplementary Table S2). The *miR-430* recognizing sites were enriched in the 3′UTR of up-regulated genes but depleted in the down-regulated genes (Figure 4B).

We next analyzed the differentially expressed genes by GO enrichment analysis. The up-regulated genes were enriched in the catabolic processes whereas the down-regulated genes were enriched in the regulation of development (Figure 4C). Genes involved in the epiboly (*pou3f1*), embryonic pattern (*chd*, *gsc*, *flh*, *zic3*, *spl5*, and *tcf7l1*) and germ layer specification (*gata5*, *snail1a*, *bon*, *sox17*, and *sox32*) were down-regulated whereas genes involved in the regulation of cell movements (*sdf-1* and *cap1*) were increased in the *miR-430*^–/–^ mutants.

Nodal signaling plays a critical role in the induction of mesendoderm cell fate specification (Schier and Talbot, 2005). Consistent with the reported functional roles of *miR-430* in the regulation of nodal signaling, we found that the down-stream genes of the nodal pathway (e.g., *chd* and *gata5*) were decreased and mesendoderm development was disrupted in the mutant. The expression level of the nodal antagonist, *lft2*, was markedly increased in the *miR-430*^–/–^ mutants (Figures 4D,E). We tested whether knockdown of *lft2* could rescue some phenotypes of the mutants. *Lft2* knockdown rescued endoderm development and the cardiac bifida (Figure 4F). These data indicate that hundreds of targets are regulated by *miR-430* and regulation of nodal signaling by *miR-430* is required for endoderm development.

### *MiR-430* Is Required for MZT

**FIGURE 5:**
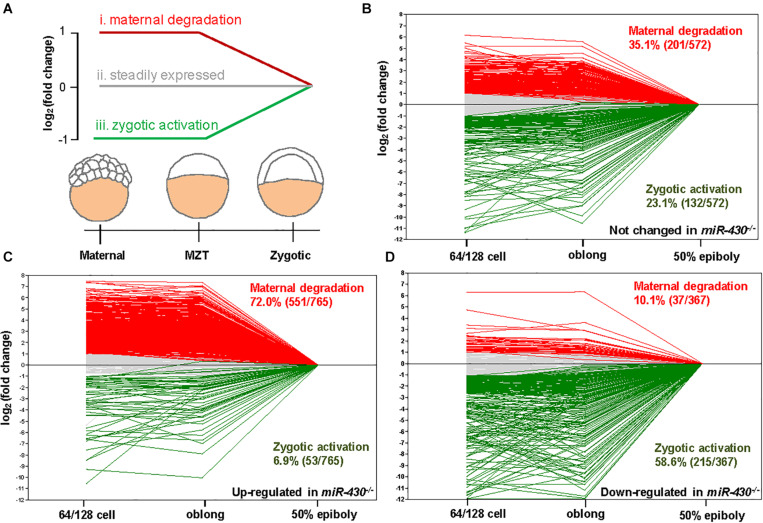
*miR-430* is required for the degradation of maternally provided transcripts and activation of zygotic gene expression. **(A)** Schematic representation of the expression profiles of *miR-430*-regulated genes during MZT (64/128-cell stages, oblong and 50 epiboly). The *miR-430*-regulated genes were grouped into three categories based on their expression profiles. The expression profiles of genes in the control set **(B)**, up-regulated set **(C)**, and down-regulated set **(D)** of the *miR-430* mutants during MZT.

Using the MZdicer embryos, previous studies suggested that *miR-430* is required for clearance of maternally provided transcripts (Giraldez et al., 2006; Lee et al., 2013). We further explored this hypothesis using the transcriptome data (Supplementary Table S2). We grouped the *miR-430*-regulated genes into three categories: (i) maternally provided transcripts that are rapidly degraded after ZGA; (ii) the steadily expressed genes during early development; and (iii) the up-regulated genes after ZGA (Figure 5A). Compared to the control data set (genes not significantly changed in the *miR-430*^–/–^ mutants), the up-regulated genes in the *miR-430*^–/–^ mutants were significantly enriched in category (i) (*p* = 3.0147E-41, Chi-squared test) but depleted in category (iii) (*p* = 2.6503E-17, Chi-squared test) (Figures 5B,C), supporting that *miR-430* is required for the clearance of maternally provided transcripts. Conversely, the down-regulated genes in the *miR-430*^–/–^ mutants were significantly enriched in the category (iii) (*p* = 7.1046E-18, Chi-squared test) but depleted in category (i) (*p* = 3.8758E-28, Chi-squared test) (Figure 5D), demonstrating that the zygotic genome expressed genes were not fully activated in the *miR-430*^–/–^ mutants. These data indicate that hundreds of maternally provided transcripts were not timely eliminated and ZGA was impeded upon loss-of-function of *miR-430*, demonstrating a critical role for *miR-430* during MZT (Supplementary Figure S5).

### A Negative Reciprocal Loop for *miR-430* in the Clearance of Maternal Transcripts

**FIGURE 6:**
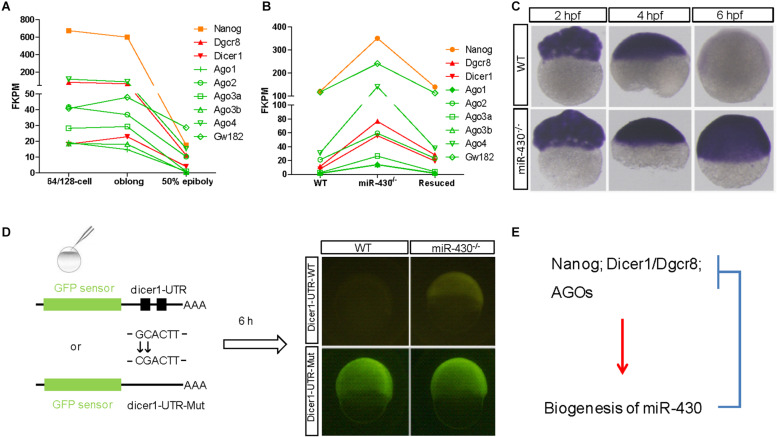
A reciprocal regulatory loop for *miR-430* in the clearance of maternal transcript. **(A)** The transcripts of *Nanog*, *Dicer1*, *Dgcr8*, and *AGOs* were maternally provided. **(B)** The maternally provided transcripts *Nanog*, *Dicer1*, *Dgcr8*, and *AGOs* were significantly increased in the *miR-430*^–/–^ mutants and down-regulated in the rescued embryos at shield stage. **(C)** WISH detection of *Dicer1* transcripts in the WT and *miR-430*^–/–^ mutants. The Dicer1 transcripts were maternally provided and degraded at shield stage in the WT but not *miR-430*-deficient embryos. **(D)** Repression of *Dicer1* by *miR-430* thorough *miR-430* binding sites in their 3′UTR. Two *miR-430* binding sites (GCACTT) were identified in the 3′UTR of *Dicer1*. GFP sensors with *Dicer*-3′UTR-WT and *Dicer*-3′UTR-Mut (two-nucleotide mutations in the *miR-430* binding sites) were injected into embryos and fluorescence was examined at 6 hpf. **(E)** A proposed reciprocal regulatory loop between maternally provided transcripts and *miR-430*.

The nature of these *miR-430*-regulated maternally provided transcripts was less explored. We found that *Nanog*, *Dicer1*, *Dgcr8*, and *AGO* transcripts are maternally provided transcripts that were degraded rapidly after ZGA (Figures 6A,C). Interestingly, these maternally provided transcripts were significantly increased in the *miR-430*^–/–^ mutants (Figures 6B,C and Supplementary Figure S6), suggesting that *miR-430* is required for the clearance of these maternally provided transcripts which were actually required for the activation, processing and function of *miR-430*. *MiR-430* binding sites were identified in the 3′UTR of these genes except for *Nanog* (Supplementary Table S3), suggesting that *miR-430* may directly regulate these transcripts through these binding sites. We have tested one of the targets, Dicer1, which possesses two *miR-430* sites in its 3′UTR (Figure 6D and Supplementary Figure S7). We found that fluorescence signal from the GFP sensor with WT UTR (GFP-Dicer1-3′UTR-WT) was suppressed in the WT embryos but not in the mutant embryos (Figure 6D). Moreover, mutation of the *miR-430* binding sites abolished the *miR-430* mediated Dicer1 suppression (Figure 6D). These data indicate that the existence of negative regulatory loop between the maternally provided transcripts and *miR-430*: that the maternally provided transcripts are required for *miR-430* biogenesis, whereas *miR-430* is required for the clearance of these maternally provided transcripts (Figure 6E).

## Discussion

In contrast to transcription factors, most gene knockout studies of miRNA produced subtle phenotypes (Alvarez-Saavedra and Horvitz, 2010). In this study, we demonstrated that genetic deletion of the *miR-430* cluster leads to embryonic lethal phenotypes with severe developmental defects. Although such a large deletion may disrupt genomic topological associated domains, but the consequence of these disruptions are not clear. The mutated phenotype can be well-rescued by *miR-430*, indicating that the large deletion event may not directly contribute to the observed phenotypes. Some rescued embryos could even survive to adulthood, indicating that *miR-430* is mainly functional during early embryonic development. Moreover, most cell types are specified in the *miR-430*^–/–^ mutants, but the myeloid progenitor cells failed to specify, suggesting that *miR-430* is essential for specific cell type emergence.

Consistent with the fact that *miR-430* could partially rescue MZ*dgcr8* and MZ*dicer* phenotypes, genetic deletion of *miR-430* generates similar phenotypes as the MZ*dgcr8* and MZ*dicer* in several aspects: developmental delay, affected cardiovascular and neural systems as well as germ layer specification and cell movement defects. Cardiac bifida and tail blister was observed in both the *miR-430*^–/–^ and MZ*dgcr8* mutants but not in the MZ*dicer*1 mutants. These phenotypes were also observed in other mutants with disrupted endoderm development and sphingosine-1-phosphate (S1P) signaling pathway (Reiter et al., 2001; Kawahara et al., 2009). In zebrafish, mutations in the S1P transporter (*spns2*), the S1P receptor (*slpr2*) and *G*α*13* disrupt myocardial migration, leading to cardiac bifida (Kupperman et al., 2000; Kawahara et al., 2009; Ye and Lin, 2013). Interestingly, *miR-430* binding sites were predicted in the 3′UTR of both *spns2* and *slpr2*. Whether *miR-430* could directly regulate these targets awaits further investigation.

miRNAs have been proposed to be required for maternal RNA clearance across species (Giraldez et al., 2006; Bushati et al., 2008; Lund et al., 2009). We tested this hypothesis using the *miR-430*^–/–^ mutants in this study. Hundreds of maternal mRNAs were not timely degraded in the *miR-430* deficient embryos. Moreover, many zygotic genome-expressed genes are not fully activated in the *miR-430*^–/–^ mutants. The prolonged presence of maternally provided transcripts may prevent full ZGA. A similar phenomenon was reported when the m6A-dependent RNA decay pathway was disrupted in zebrafish embryos, in which the maternally provided transcripts were not timely degraded and ZGA was impeded (Zhao et al., 2017). These data demonstrate that *miR-430* is not only required for the clearance of maternal RNA but also contribute to ZGA.

In this study, we found that hundreds of maternally provided transcripts are regulated by *miR-430*. One interesting observation is that *Nanog*, *Dicer1*, *Dgcr8*, and AGOs, which are required for transcription, processing and targeting of *miR-430* were regulated by *miR-430*, suggesting that a reciprocal regulatory loop existed between the maternal transcripts and *miR-430*. By analysis of gene enrichment in a particular pathway, we further found that many of the *miR-430* targeted maternally provided transcripts are involved in the catabolic processes (Figure 4C), suggesting that these maternally provided transcripts may play important roles in controlling their metabolism in the embryo. After ZGA, the *miR-430* was produced and these maternally provided transcripts were degraded, thus *miR-430* may facilitate a shift in metabolic state during MZT.

## Conclusion

In summary, the translation of maternally provided transcripts promotes *miR-430* biogenesis shortly after fertilization and the synthesized *miR-430* in turn promotes termination of the maternal program by inducing degradation of the maternally provided transcripts. Moreover, *miR-430*s also promote the zygotic program by regulating developmental pathways that are required for cell movement, germ layer specification, axis patterning and organ progenitor formation in the embryos. These results demonstrate that *miR-430* is required for proper degradation of maternally provided transcripts and impact the later embryonic development.

## Data Availability Statement

NCBI Sequence Read Archive (SRA) reference number: SRX5707650 is the reference series for our publication: https://www.ncbi.nlm.nih.gov/sra/SRX5707650.

## Ethics Statement

The animal study was reviewed and approved by Animal Experimentation Ethics Committee of the Chinese University of Hong Kong.

## Author Contributions

YL, IH, and ZZ analyzed the genotypes and phenotypes. YL, YS, and ZZ carried out marker gene analysis. YL, JL, and XW performed transcriptome experiments. YL, MC, and CC conceived the research and designed the experiments and wrote the manuscript. All authors contributed to the article and approved the submitted version.

## Conflict of Interest

YL was employed by the company Shenzhen Zelong Biological Technology Limited Cooperation. The remaining authors declare that the research was conducted in the absence of any commercial or financial relationships that could be construed as a potential conflict of interest.

## Footnotes

1 http://www.targetscan.org/fish_62/

2 https://david.ncifcrf.gov/

## Abbreviations

FPKM: fragments per kilobase million
hpf: hours post fertilization
lft2: lefty2
MZdgcr8: maternal zygotic dgcr8
MZdicer: maternal-zygotic dicer
MZT: maternal zygotic transition
UTR: untranslated region
ZGA: zygotic genome activation.
