# Supplementary material for: Genetic Deletion of miR-430 Disrupts Maternal-Zygotic Transition and Embryonic Body Plan
Source: Front Genet. 2020 Aug 4;11:853. doi: 10.3389/fgene.2020.00853 (PMC7417628; doi:10.3389/fgene.2020.00853)
Supplement: Supplementary file 1 [file Data_Sheet_1.doc]

**
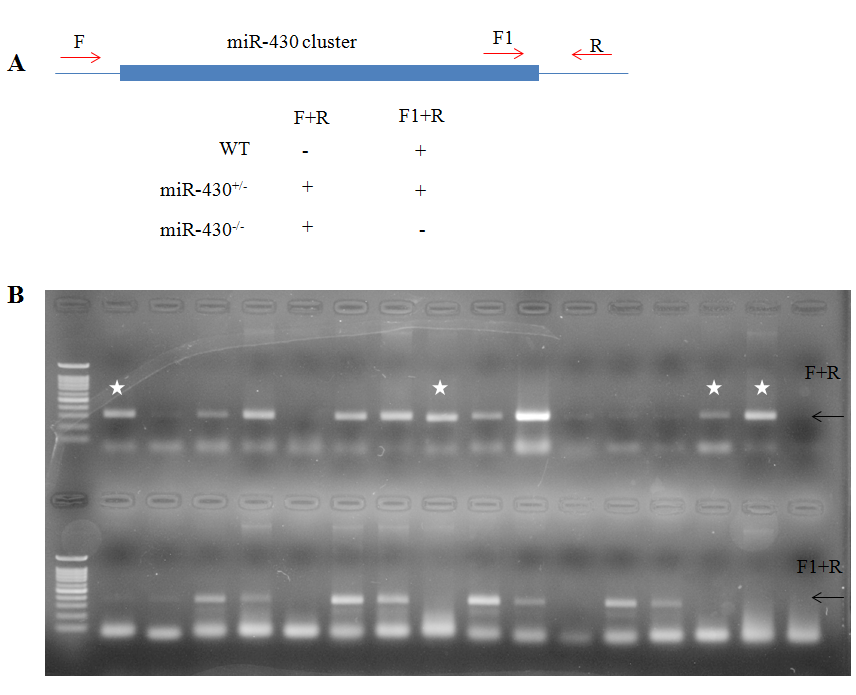
**

**Figure S1** **Genotyping of *miR-430* mutants.** (A) The position of primers used for *miR-430* genotyping. To differentiate each genotype (WT, *miR-430*^+/-^ and *miR-430*^-/-^), two pairs of primers were used. (B) Genotyping of individual *miR-430* embryo produced by incrosses of *miR-430* heterozygote adults. The *miR-430* homozygotes were indicated by stars.

**Figure S2**

**Figure S2** **Morphology of *miR-430***^-/-^ **mutants at 2 d.p.f. and 5 d.p.f..** (A) Morphology of WT and *miR-430*^-/-^ mutant at 2 d.p.f. The body axis was shorter and curved in the mutant. (B) Morphology of WT and mutant at 5 d.p.f. The body cavity was swollen in the *miR-430*^-/-^ mutant.

**Figure S3**

**
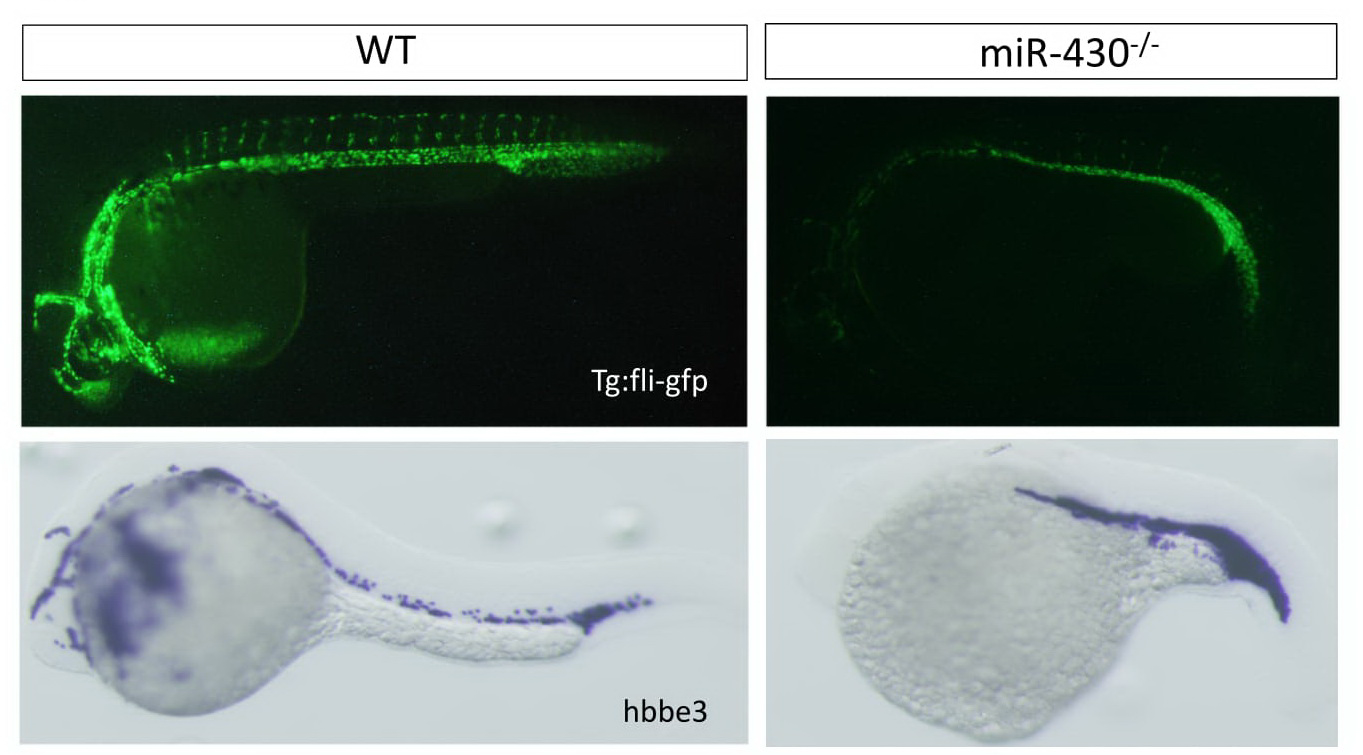
**

**Figure S3** **Circulation system of *miR-430***^-/-^ **mutant at 26 h.p.f..** The vasculature system of the WT and the *miR-430*^-/-^ mutant at 26 h.p.f.. The vasculature was analyzed using the Tg:(fli-EGFP);*miR-430*^-/-^ line. The segmental vessels were disrupted in the *miR-430*^-/-^ mutant. The erythrocytes (marked by *hbbe3*) were accumulated at the aorta-gonad-mesonephros region in the *miR-430*^-/-^ mutant.

**Figure S4**

**Figure S4** **Validation of transcriptome data using Q-PCR.** (A) The expression of the tested genes in the transcriptomes. (B) The relative expression of the selected genes measured by Q-PCR. The transcript levels were normalized against that of WT control. Embryos were collected at shield stage. Data are expressed as mean values ± S.E.M (n = 4).

**Figure S5**

**
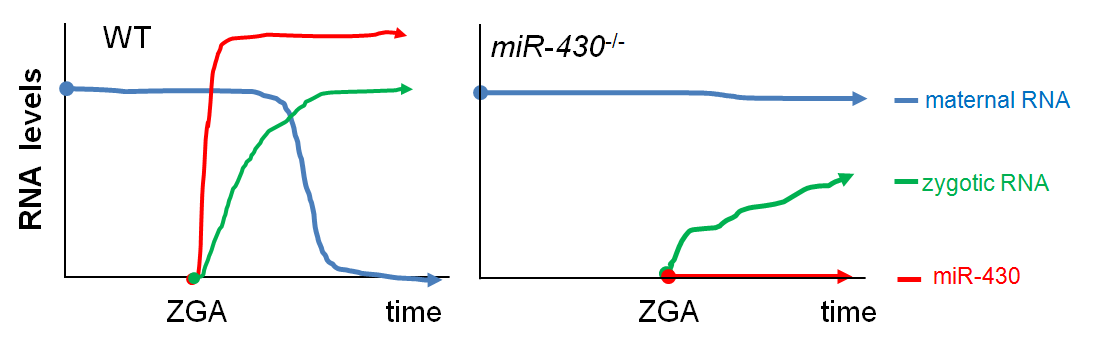
**

**Figure S5** **A schematic model for *miR-430* function during MZT**. In the WT embryos, the MPTs promote *miR-430* biogenesis and *miR-430* induces the clearance of MPTs, and the zygotic genome expressed genes are fully activated. In the *miR-430*^-/-^mutant, the MPTs are not timely degraded and the zygotic genome expressed genes are not fully activated.

**Figure S6**

**
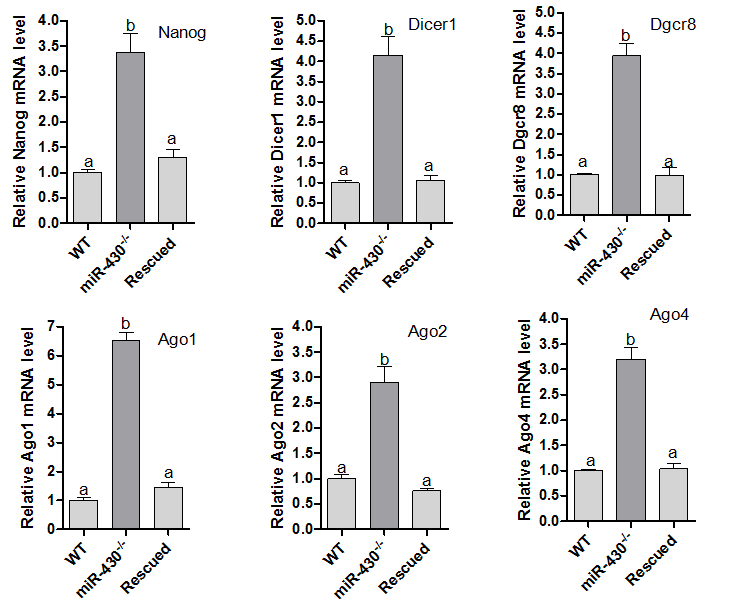
**

**Figure S6** **Relative expression of *Nanog*, *Dicer1*, *Dgcr8*, *Ago1*, *Ago2* and *Ago4* in the WT, *miR-430*^-/-^ and rescued embryos.** Embryos were collected at shield stage. Gene expression was analyzed using Q-PCR. Data are expressed as mean values ± S.E.M (n = 4). Significant differences among groups are indicated by different letters.

**Figure S7**

>3’UTR of zebrafish *Dicer1*

CAGGTCCAAAACAAC**TGA**GACACCCGGTCAAATACGCCGCAATGAACCAATCCAGTCACGGGATTTCAGCTCAATTCACATGGGGTTCATATTTCATGTTATTCTTTACGTGCGCAAACACAATCTTTTTTTCTCCCCAGCTTTGTTGGATCACGAGTGCTTTTCATGTATCGCAAGTGTTAAGAAAAAGGTCAGGTCTTCTTACACCCACAATTTCCCTATTTTTATTAACATTTAAAAAAAGAAAGATATATGCTCTGCTTCGTAGTAGTGGTTAAAATAGCTAAAGCTTATAGGTAGTATTACAGTGTACATAGTTCATAACTCCAAGCTATCAACTAAGTAAGAGAAATAAAAAAAAAGTGACATACAAACACAAAATTCCTCAATCTGTGCACTAGTGCCAGGAAGGGCACGTAAGATGGTGTTGGTATTGGAAGTGTTTTTATCTTGAGATGACTTTACTCTCAATCAAACTTGCACTTCTGTAGGGTCCGTTTTATTAGGTAGTTTTTTTTTTTCCGCCAGATCACAGCGGAATCGTTTTAGATCTATATTGTATTGAAAGTGAAAGGCCCTTGCATCCTTGAGACAGATAGGAATTTGACATTCCCGATTACTGTAATCAGCATAGTTGTGAACGGCACTTGAGGATGCATGTTCGTGTAGCCAACCGGATGGGTTGTATAGACGTCATAAA

**Figure S7** **The 3′UTR sequence of *Dicer1*.** The 3′UTR of *Dicer1* (*Dicer*-3′UTR-WT) was amplified from WT embryos using primers (underlined) listed in Table S1. The stop codon was shown in bold and 3′UTR was shown in red. Two miR-430 binding sites were boxed in yellow. The mutated 3′UTR of Dicer1 (*Dicer*-3′UTR-mut) was synthesized and both miR-430 binding sites were mutated from GCACTT to CGACTT.

**Table S1 Primers used in this study**

| **Primer name** | | **Sequence (5′-3′)** | | **Purpose** | |  |
| --- | --- | --- | --- | --- | --- | --- |
| miR-430-F | | CACATATTGATCATTACTGCTAAC | | Genotyping of *miR-430* mutants | |  |
| miR-430-F1 | | GTCCCGATAGACTCTGCTAGAG | |  | |  |
| miR-430-R | | CTCGCAGATTGGAATCTATCCTTC | |  | |  |
| miR-430-pF | | TCGAATTCGCTAAAGACTATGAACGGACAAGG | | PCR amplification of pri-mir-430 for probe synthesis | |  |
| miR-430-pR | | AGCTCGAGGTGGGCAGAGGTGACTAAGTCAG | |  |
| ago3b-qPCR-F | | CCTACTATGCTCATCTGGTG | | Q-PCR amplification of zebrafish *ago3b* | |  |
| ago3b- qPCR-R | | GTGATTGACAGGTCTGTGGAG | |  |
| pycr1b-qPCR-F | | CAGCGAAGATGTTGTTGGAC | | Q-PCR amplification of zebrafish *pycr1b* | |  |
| pycr1b-qPCR-R | | CACAGCGTTGATCAGCAAGC | |  |
| map4k5-qPCR-F | | GTCACACAGGAGATTTCAGAC | | Q-PCR amplification of zebrafish *map4k5* | |  |
| map4k5-qPCR-R | | CAGGATGTAGAGGTTACTG | |  |
| pcxa-qPCR-F | | GTGAAGCAAGGGCAGCAAGTG | | Q-PCR amplification of zebrafish *pcxa* | |  |
| pcxa -qPCR-R | | GTGCTGTCGGCGGTCACGTAG | |  |
| tmem-qPCR-F | | TAGACACAGTCCAGTTCGCC | | Q-PCR amplification of zebrafish *tmem* | |  |
| tmem -qPCR-R | | TGTGGGGTTGTAGGGAGAGT | |  |
|  | foxi1-qPCR-F | | CGACGGAAACGCGATGTCTGTG | | Q-PCR amplification of  zebrafish *foxi1* | |
|  | foxi1-qPCR-R | | GAGAGGATTTGGGATCGCTG | |
|  | nip7-qPCR-F | | GATCGGAGCAATCATTCCTG | | Q-PCR amplification of zebrafish *nip7* | |
|  | nip7-qPCR-R | | GTGTTGTTCTCGCTGCAACTC | |
|  | znfl2a-qPCR-F | | GAGTGAAGAGGACAGCG | | Q-PCR amplification of zebrafish *znfl2a* | |
|  | znfl2a -qPCR-R | | CTGACGGCAGCTCCACGTC | |
|  | ssr1-qPCR-F | | CGTCTTCAACCAGACTGTCAC | | Q-PCR amplification of zebrafish *ssr1* | |
|  | ssr1-qPCR-R | | ACTTTAGCTGCAGGCCGTCTC | |
| gata5-qPCR-F | | CACTTCACTGCCTGTGTCAG | | Q-PCR amplification of zebrafish *gata5* | |  |
| gata5-qPCR-R | | CACATGGGCAGAACTGGCAC | |  |
| lft2-qPCR-F | | CATGGCTCTGTTCATCCAGCTG | | Q-PCR amplification of zebrafish *lft2* | |  |
| lft2-qPCR-R | | TGGGCGGTTCAGTTAGTCCGAG | |  |
| dicer1-qPCR-F | | CTCCTGGAGATGGAGCCAG | | Q-PCR amplification of zebrafish *dicer1* | |  |
| dicer1-qPCR-R | | CTATCCGGTAGCTGCGTCCTAC | |  |
| dgcr8-qPCR-F | | GACGGTGTTACATACGGCACTG | | Q-PCR amplification of zebrafish *dgcr8* | |  |
| dgcr8-qPCR-R | | GATTTCCAGTGTTGCTCGAG | |  |
| ago1-qPCR-F | | GCATACTACGCCAGACTCGTG | | Q-PCR amplification of zebrafish *ago1* | |  |
| ago1-qPCR-R | | GGGAGTCGTGGTGAATCTGCAC | |  |
| ago2-qPCR-F | | CACTATCATGTGCTGTGGGAC | | Q-PCR amplification of zebrafish *ago2* | |  |
| ago2-qPCR-R | | GTTACTCTGACCTGATGTGTG | |  |
| ago4-qPCR-F | | GACCTATCAGCTCTGCCACAC | | Q-PCR amplification of zebrafish *ago4* | |  |
| ago4-qPCR-R | | TGTCGTAGTGAATCTGCACAG | |  |
| nanog-qPCR-F | | TTGTTAAGCTGAGCGAAGCAGAC | | Q-PCR amplification of zebrafish *nanog* | |  |
| nanog-qPCR-R | | TGTGCTTGCTGTTCTTGGTCAC | |  |
| dicer-UTR-XhoIF | | AGACTCGAGCAGGTCCAAAACAACTGAGAC | | PCR amplification of zebrafish UTR of *dicer1* | |  |
| dicer-UTR-XbaIR | | GATCTAGACGTCTATACAACCCATCCGGTTG | |  |
| chdF | | GTCGTGAGCTCTTCTGGTTGTC | | For *chd* probe synthesis | |  |
| chdR | | TCGTAATGCAGATGACACTGAG | |  | |  |
| gscF | | CTTGGCCGGGAGACCCAGCTG | | For *gsc* probe synthesis | |  |
| gscR | | TCAGCTGTCAGAATCCACGTC | |  | |  |
| bmp4F | | AGAAAGCAGCTATGCTAGTCTG | | For *bmp4* probe synthesis | |  |
| bmp4R | | CGACTTACACGAACATGTCGTC | |  | |  |
| eve1F | | CACCAGTGTCTACTGTTGCTC | | For *eve1* probe synthesis | |  |
| eve1R | | TACTGGATGATGCATTGTCTG | |  | |  |
| snailF | | CTAGCAGAGCTTCCAGCAGTCAG | | For *snail* probe synthesis | |  |
| snailR | | GCTGCAGCAGTGACATGCGACTG | |  | |  |
| mixer1F | | CTCCAGGGCAGACAACGTCAG | | For *mixer1* probe synthesis | |  |
| mixer1R | | GAGTCAGAATCGGAGAACTCGAC | |  | |  |
| sox17F | | GTACTCCAGTGACGATCCGAG | | For *sox17* probe synthesis | |  |
| sox17R | | CAATGCTGTGAGACTGTCTG | |  | |  |
| sox32F | | GACATATTCATTTATTTACATC | | For *sox32* probe synthesis | |  |
| sox32R | | CAAATGCCACCTTTGATTTCAG | |  | |  |
| nkx2.5F | | GACTTCCACTCCTTTCTCAGTG | | For *nkx2.5* probe synthesis | |  |
| nkx2.5R | | TAGTTGCATGAGTAGTTCGAG | |  | |  |
| pu.1F | | CTGCATCCGTACAGAATGGAG | | For *pu.1* probe synthesis | |  |
| pu.1R | | CAGTGCTCTTGCCATCTTCTG | |  | |  |
| gata1F | | GAACTCCTCTGAGCCTTCTC | | For *gata1* probe synthesis | |  |
| gata1R | | CAGGTGGCGAAAGTCTCATC | |  | |  |
| fli1F | | ACGTGCGGCAGTGGTTAGACTG | | For *fli1* probe synthesis | |  |
| fli1R | | CAAGGTGTGAAGGCACGTGTG | |  | |  |
| six3bF | | CAGCGTGTGCGAGACGCTGGAG | | For *six3b* probe synthesis | |  |
| six3bR | | TCTGACATGGAGCGCAGACTG | |  | |  |
| eng2F | | GTACTCCCTCACAGAATCACC | | For *eng2* probe synthesis | |  |
| eng2R | | TCTTGAGTCTCTGTAGTTGCTC | |  | |  |
| kox20F | | GAGTCTCGGTGGCTTTGTGCAC | | For *kox20* probe synthesis | |  |
| kox20R: | | GCGTGAAGTTCCTGATAGTG | |  | |  |
| pax2aF | | GAGTCTCGGTGGCTTTGTGCAC | | For *pax2a* probe synthesis | |  |
| pax2aR | | GTGTTCCTGTGCCGTCGGAG | |  | |  |
| cmlc2F | | GAACAAATGATAAGAGTCCTC | | For *cmlc2* probe synthesis | |  |
| cmlc2R | | CACGTCTATTGGAGCCACTG | |  | |  |

**Table S2 *MiR-430*-regulated genes.**

**Table S3 Identification of *miR-430* binding sites in the 3′UTR of genes in the miRNA pathway**

| **Genes** | **8mer** | **7mer-m8** | **7mer-1A** | **6mer** | **# of total sites** |
| --- | --- | --- | --- | --- | --- |
| *dicer1* | - | - | - | 2 | 2 |
| *dgcr8* | - | - | - | 1 | 1 |
| *ago1* | - | - | 1 |  | 1 |
| *ago2* | - | - | - | 2 | 2 |
| *ago3a* | 1 | - | - | 1 | 2 |
| *ago3b* | 2 | - | - | - | 2 |
| *ago4* | - | - | - | 1 | 1 |
| *gw182* | 1 | - | - | - | 1 |
